# Game load dynamics in basketball: influence of playing position and match progression

**DOI:** 10.3389/fphys.2026.1713000

**Published:** 2026-02-06

**Authors:** Sebastián Feu, Juan Manuel García-Ceberino, Pablo López-Sierra, Sergio José Ibáñez

**Affiliations:** 1 Facultad de Educación y Psicología, Universidad de Extremadura, Badajoz, Spain; 2 Facultad de Ciencias del Deporte, Universidad de Extremadura, Cáceres, Spain

**Keywords:** external load, internal load, inertial devices, competition, linear mixed model

## Abstract

**Objectives:**

This study explored the variability of internal and external load in professional basketball as a function of playing position and match quarter.

**Methods:**

Fourteen ACB League players were monitored across three official games using ultra-wideband tracking and inertial measurement units (113 observations). Linear mixed-effects models assessed the influence of match progression and positional roles on kinematic, neuromuscular, and physiological load variables.

**Results:**

External load indicators, specifically total distance covered (*p* < 0.001; *ηp* ^*2*^ = 0.172), neuromuscular load (*p* < 0.001; *ηp* ^*2*^ = 0.178), and high-intensity actions (*p* = 0.038; *ηp* ^*2*^ = 0.030), declined progressively from the first to later quarters, confirming that the opening quarter imposes the highest physical demands. Guards performed more high-intensity actions per minute than centers (*p* = 0.045; *ηp* ^*2*^ = 0.473). High inter-individual variability across several variables further supported the need for individualized monitoring.

**Conclusions:**

These findings emphasize the importance of considering both match progression and positional roles when designing training programs. Understanding how load fluctuates across quarters could help coaches optimize periodization, adjust substitution strategies, and develop conditioning plans that had better reflect the repeated high-intensity efforts required in professional basketball.

## 1 Introduction

Load monitoring and analysis, both during training and competition, has become a key component of preparation in high-performance sports (Feu et al., 2023; García et al., 2022). In intermittent sports such as basketball, where physical demands vary considerably and depend on multiple contextual factors (López-Sierra et al., 2025; Sansone et al., 2021), understanding the load borne by each player is crucial for adequately planning sessions, facilitating effective recovery, and reducing the risk of injuries associated with excessive or poorly distributed loads (Fox et al., 2017). To achieve this, it is not only necessary to have evidence regarding the average values recorded during competition (Conte et al., 2022), but also about the peak demand scenarios that players face throughout the games (García et al., 2022).

The monitoring and quantification of the intensity to which players are exposed, commonly referred to as load demands, is one of the most important aspects of training and competition management (Staunton et al., 2022; Scanlan et al., 2014; García-Cuevas et al., 2025). An effective approach to this analysis involves differentiating between external and internal load. External load (eTL) refers to the physical stimuli imposed on the player and can be measured objectively using technologies such as accelerometers, positioning systems, or GPS tracking devices (e.g., total distance covered, number of sprints, or accelerations (Gómez-Carmona et al., 2020). These measurements are particularly relevant given that basketball is a contact sport characterized by frequent accelerations and decelerations (Wellm et al., 2024). Conversely, internal load (iTL) refers to the individual’s physiological or perceptual response to those stimuli, which can be assessed using indicators such as heart rate or rating of perceived exertion (Scanlan et al., 2014; Gómez-Carmona et al., 2020). The complementary use of both perspectives allows for a more accurate and context-specific understanding of the actual effort sustained by the athlete.

In the competitive context, this analysis becomes even more relevant. The demands of actual gameplay often differ substantially from those encountered in training, not only in terms of intensity and duration but also in tactical complexity (Feu et al., 2023). Therefore, monitoring load during official matches serves not only to verify whether training appropriately reflects the demands of competition but also to assess whether load is adequately individualized. Furthermore, being a team sport, basketball load can be influenced by factors such as the opponent, the phase of the match, or the player’s tactical role, highlighting the need for detailed and context-aware monitoring. Load demands may vary depending on the game quarter (Miró et al., 2024; García et al., 2020; Salazar et al., 2024; Pérez-Chao et al., 2022; Fox et al., 2021).

Accordingly, conducting inferential analyses that account for players’ specific positions becomes essential. Not all roles within the game involve the same physical demands (Vázquez-Guerrero et al., 2018; Sanchez-Castillo and Pons, 2022; Stojanovic et al., 2018; Svilar et al., 2018). For instance, point guards typically accumulate more high-speed actions, changes of direction, and short-distance movements, whereas centers are often subject to increased physical contact, isometric efforts, and strength-based actions near the basket (Miró et al., 2024; Ibanez et al., 2024). Ignoring such differences can lead to inaccurate interpretations or suboptimal training plans that do not reflect the actual needs of each player profile. Position-specific statistical analysis allows for the identification of significant differences between roles, enabling more precise planning aligned with the specific demands of the game.

Ultimately, integrating both iTL and eTL analysis within the competitive context, while also considering positional differences, supports more informed decision-making in physical preparation. This approach not only enhances performance but also contributes to more effective load management across the season, with the aim of preserving athlete health and sustaining long-term performance (Sánchez et al., 2019; Tuttle et al., 2024). The aim of this study was to analyze the variables that determine physical exercise demands in professional basketball, considering both the context of measurement (i.e., the game quarter during official competition) and the specific playing position of each athlete.

## 2 Materials and methods

### 2.1 Study design

An associative methodological strategy was adopted, with a comparative and cross-sectional design (Ato et al., 2013). The study was conducted in the natural sports environment, with no intervention by the research team in the development conditions, following an *ex post facto* analysis approach (Montero and León, 2007).

### 2.2 Participants and sample

Fourteen professional players from the Spanish first division basketball league were monitored during three competitive microcycles in the 2022–2023 season. The sample consisted of each player’s participation in each quarter of the competition. A total of 113 records were analyzed from three official matches. Thirty-two variables were collected, resulting in a data matrix of 3,616 cases. Research examining coaches’ behaviors in team-sport settings frequently relies on relatively small samples. Nevertheless, this does not diminish the validity of the knowledge produced or its capacity to contribute meaningfully to the scientific community. In this context, our study aligns with the criteria proposed by Lago et al. (2020) for generating robust scientific evidence: (1) supplementing traditional significance testing with magnitude-based metrics (such as effect sizes); (2) increasing the number of observations whenever feasible; and (3) striving to develop explanatory principles of players’ behavior based on Merton’s middle-range theory.

### 2.3 Variables

From the total variables collected in the study, Table 1 includes the iTL and eTL variables, considered as dependent variables. All variables were normalized per minute.

**TABLE 1:** Load demand variables used in the study.

Demand type	Variable	Abbrev.	Description
Kinematic eTL	Distance/min	DIST/MIN	Distance covered in meters per minute
	High-intensity actions	HIA/MIN	Number of high-demand actions per minute. This is the sum of the following variables: Take offs (>3G), landings (>5G), impacts (>8G), accelerations (>3 m/s^2^), decelerations (<-3 m/s^2^), relative sprints (>95% max speed), and relative HSR (>75.5% max speed)
	High G actions	HGA/MIN	Actions involving a G-force greater than 8G
	Accelerations	ACC+2/MIN	Number of accelerations >2 m/s^2^ per minute
	Decelerations	DEC+2/MIN	Number of decelerations < -2 m/s^2^ per minute
	Acc.-Dec. Difference/min	ACC-DEC/MIN	Mean difference between high accelerations and decelerations (>3 m/s^2^) per minute
	Jumps/min	J+3G/MIN	Number of take-offs and jumps per minute. These involve a flight time >400 m
	Landings/min	L+5/MIN	Number of landings per minute
Neuromuscular eTL	Impacts	IMP+8/MIN	Number of impacts per minute, defined as the vector sum of G-forces sustained in the x, y, and z planes
	Player Load/min	PL/MIN	Vector magnitude derived from triaxial accelerometry data
iTL	Average heart rate	HRAVG	Arithmetic mean of heart rate in beats per minute
	Max heart rate	HRMAX	Arithmetic mean of the maximum heart rate in beats per minute

eTL, external load; iTL, internal load.

Intensity-related metrics were derived using two complementary approaches. First, the High-Intensity Actions (HIA) variable integrated several mechanically demanding events automatically detected by the WIMU-PRO system, including take-offs (>3G), landings (>5G), high-intensity impacts (>8G), accelerations and decelerations above the device-defined thresholds, and speed-based actions. High-speed running (HSR) was identified when players exceeded 75.5% of their individual maximal in-game speed, while sprinting was defined as movement above 95% of maximal speed. All HIA events were subsequently normalised per minute of effective playing time to quantify the frequency of high-demand actions.

Second, accelerations and decelerations were also examined as independent kinematic variables. These were defined as the number of occurrences exceeding a fixed threshold of ±2 m s^-2^. As count-based measures, no minimum dwell time was required, and events were detected at the device’s 100 Hz sampling frequency. This threshold enables the identification of meaningful changes in speed within the movement patterns typical of basketball play.

For statistical analysis, specific positions were recoded into three groups: point guards, perimeter players (shooting guards, small forwards), and interior players (power forwards, centers).

### 2.4 Instruments

Data collection took place in indoor facilities using IMU technology with ultra-wideband (UWB) tracking at 100 Hz, to record distances covered at various speeds, as well as accelerations and decelerations. This monitoring system has shown high reliability and validity for indoor data collection (Bastida-Castillo et al., 2019). Eight portable radiofrequency antennas were installed and interconnected following the same protocol used in similar studies (Reina et al., 2022; Ibáñez et al., 2023). In that way, prior to the data-collection, inter-device reliability was assessed by walking the perimeter of the court while carrying two WIMU-PRO units attached together. This procedure ensured optimal signal quality and confirmed that both units recorded data consistently at the expected sampling frequency (100 Hz). After data extraction, the inter-device distance was examined using rolling averages calculated along the entire perimeter trajectory. A mean absolute error below 7 cm was required to verify minimal measurement error across the playing surface. When deviations greater than 7 cm were detected, the antenna configuration was reassembled and the validation protocol repeated until optimal reliability was achieved.

Each athlete was equipped with a GARMINTM heart rate pectoral band (Garmin Ltd., Olathe, KS, United States), and a WIMU-PROTM inertial device. The heart rate band was synchronized to the inertial unit via ANT + protocol. Data were collected using S-PROTM software (RealTrack Systems, Almería, Spain; now part of Hudl, United States) and later exported to statistical software for analysis.

To measure impacts, accelerations, decelerations, jumps, and landings, the inertial device incorporated several microsensors (four accelerometers: two 16 g, one 32 g, and one 400 g; three gyroscopes: 2000°/s; and one magnetometer). These microsensors operated at 100 Hz and demonstrated near-perfect validity for raw accelerometer data. Heart rate was recorded simultaneously using a chest-strap sensor operating at 1 Hz, with values expressed in beats per minute.

### 2.5 Procedure

The technical directors of the participating clubs were contacted and informed about the study’s objectives and invited to participate. Upon agreement, the rest of the coaching staff and players were informed. Ethical approval was obtained from the University Bioethics Committee (do not fill in the initial submission, do not delete in the initial submission). The study adhered to the ethical standards of the 1975 Declaration of Helsinki (as revised in later years) (Harriss and Atkinson, 2015), and to the Spanish Organic Law 3/2018, of December 5, on the Protection of Personal Data and Guarantee of Digital Rights (BOE, 294, 06/12/2018).

Before each game, eight portable antennas were installed around the court in an octagonal configuration, placed at a height of 3 m. Sixty minutes prior to tip-off, players were fitted with a tight anatomical vest positioned at the scapular level (T2–T4), into which the WIMU-PROTM device was inserted. A heart rate band was placed underneath. During competition, the S-VIVOTM software (RealTrack Systems, Almería, Spain; now part of Hudl, United States of America) was used to mark the active competition periods (start and end of each quarter). Data were not collected during inactive moments (e.g., breaks, time-outs, bench time), in order to prevent them from skewing the results.

After each match, data were downloaded to a laptop and processed using the manufacturer’s S-PROTM software to extract iTL and eTL variables. An individual performance report was generated after each training session and official game and shared with the technical staff, detailing relevant data for each athlete.

### 2.6 Statistical analysis

Descriptive statistics including mean, median, and standard deviation were calculated to characterize the load variables. Data normality was assessed using the Kolmogorov-Smirnov test (Field, 2013). Load analyses were conducted using linear mixed-effects models, with the player ID as the random factor and position and game quarter as fixed effects. Model fit was evaluated using *AIC/BIC* values and *marginal and conditional R* ^*2*^. Intraclass correlation coefficients (*ICC*) and their significance were calculated to determine whether the random subject effect was relevant. Bonferroni *post hoc* tests were used for pairwise comparisons of fixed effects. All analyses were performed using JAMOVI software.

Effect sizes were reported using partial eta squared (*partial η* ^*2*^), which quantifies the proportion of variance explained by each effect while excluding variance from other sources. The interpretation thresholds were: ∼0.01 for small effect, ∼0.06 for medium effect, and ∼0.14 for large effect.

## 3 Results

Table 2 presents the descriptive statistics of the load variables according to player position.

**TABLE 2:** Descriptive statistics of eTL and iTL by player position.

					Skewness		Kurtosis		Shapiro-wilk	
Variable	Position players	*M*	*Md*	*SD*	*Ske*	*SE*	*Kur*	*SE*	*W*	*p*
DIST/MIN	Point guards	64.992	654.25	8.092	−0.537	0.550	0.129	1.063	0.965	0.726
	Perimeter	66.788	660.21	11.854	0.536	0.369	2.513	0.724	0.954	0.094
	Interior	63.149	635.63	14.495	−0.244	0.322	0.580	0.634	0.986	0.762
HIA/MIN	Point guards	9.398	77.50	5.125	19.018	0.550	3.974	1.063	0.798	0.002
	Perimeter	6.182	60.57	2.389	12.808	0.369	4.158	0.724	0.907	0.003
	Interior	6.285	56.43	2.430	0.471	0.322	0.639	0.634	0.948	0.018
ACC2/MIN	Point guards	22.318	226.21	2.326	0.423	0.550	−0.392	1.063	0.938	0.297
	Perimeter	20.713	198.46	5.388	32.918	0.369	12.202	0.724	0.600	<0.001
	Interior	19.908	201.18	2.978	−0.861	0.322	2.036	0.634	0.960	0.062
DEC2/MIN	Point guards	22.350	220.00	2.223	0.526	0.550	−0.460	1.063	0.947	0.411
	Perimeter	20.815	198.89	5.403	32.898	0.369	12.217	0.724	0.595	<0.001
	Interior	19.975	202.35	2.977	−0.840	0.322	2.014	0.634	0.961	0.070
ACC-DEC/MIN	Point guards	44.668	445.52	4.527	0.4754	0.550	−0.427	1.063	0.943	0.351
	Perimeter	41.528	395.56	10.781	32.989	0.369	12.249	0.724	0.595	<0.001
	Interior	39.884	403.53	5.944	−0.854	0.322	2.040	0.634	0.961	0.072
J3G/MIN	Point guards	0.180	0.12	0.172	14.421	0.550	2.797	1.063	0.871	0.023
	Perimeter	0.124	0.09	0.123	0.775	0.369	−0.510	0.724	0.872	<0.001
	Interior	0.164	0.14	0.135	0.968	0.322	0.836	0.634	0.922	0.002
L5MIN	Point guards	0.217	0.14	0.155	0.368	0.550	−1.021	1.063	0.934	0.256
	Perimeter	0.222	0.21	0.193	13.732	0.369	2.582	0.724	0.892	<0.001
	Interior	0.180	0.18	0.147	0.607	0.322	−0.113	0.634	0.935	0.005
HGA/MIN	Point guards	3.032	34.44	1.354	−0.251	0.550	−0.988	1.063	0.945	0.387
	Perimeter	1.926	18.60	1.022	0.653	0.369	0.273	0.724	0.964	0.209
	Interior	1.746	14.29	1.178	0.704	0.322	−0.337	0.634	0.931	0.004
IMP8/MIN	Point guards	1.825	20.00	0.767	−0.219	0.550	−1.447	1.063	0.903	0.077
	Perimeter	1.010	0.91	0.648	12.048	0.369	1.852	0.724	0.918	0.006
	Interior	1.032	0.81	0.791	0.910	0.322	−0.153	0.634	0.898	<0.001
PL/MIN	Point guards	1.201	11.99	0.205	0.096	0.550	−1.032	1.063	0.967	0.761
	Perimeter	1.186	11.97	0.277	−11.006	0.369	6.602	0.724	0.866	<0.001
	Interior	1.123	10.75	0.282	0.051	0.322	1.094	0.634	0.968	0.146
HRAVG	Point guards	158.176	1.570.00	13.616	0.704	0.550	−0.541	1.063	0.904	0.080
	Perimeter	153.610	1.580.00	16.131	−24.051	0.369	9.200	0.724	0.782	<0.001
	Interior	146.512	1.510.00	19.767	−0.589	0.361	−0.451	0.709	0.949	0.056
HRMAX	Point guards	182.647	1.820.00	9.740	−0.156	0.550	−0.919	1.063	0.954	0.525
	Perimeter	181.829	1.830.00	15.274	−30.693	0.369	13.256	0.724	0.693	<0.001
	Interior	175.581	1.810.00	17.637	−18.456	0.361	3.719	0.709	0.805	<0.001

Dist/Min = Distance per minute, HIA/Min = High Intensity Actions per Minute, Acc2/Min = Accelerations higher than 2G per minute, Dec2/Min = Decelerations higher than 2G per minute, Acc-Dec/Min = Ratio Acceleration–Deceleration per minute, J3G/Min = Jumps over 3G per minute, L5G/Min = Landing over 5G per minute, HGA/Min = High G Actions per minute, Imp8/Min = Impacts over 8G per minute, PL/Min = Player Load per minute, HRAvg, Average Heart Rate; HRMax, Maximum Heart Rate.

In Table 3, the descriptive statistics of the load variables are presented according to the game quarter.

**TABLE 3:** Descriptive statistics of eTL and iTL based on game quarter.

					Skewness		Kurtosis		Shapiro-wilk	
Variable	*Q*	*M*	*Me*	*DE*	*Asi*	*EE*	*Curtosis*	*EE*	*W*	*p*
DIST/MIN	1	74.344	765.03	14.069	−2.172	0.441	7.845	0.858	0.821	<0.001
	2	59.811	609.47	9.852	−0.495	0.414	0.478	0.809	0.971	0.540
	3	63.565	660.54	10.186	−0.784	0.448	0.605	0.872	0.953	0.260
	4	61.712	610.10	12.066	1.747	0.456	6.396	0.887	0.857	0.002
HIA/MIN	1	7.330	74.42	2.556	−0.851	0.441	1.763	0.858	0.915	0.027
	2	6.344	55.66	2.488	1.717	0.414	3.838	0.809	0.851	<0.001
	3	7.061	56.67	4.411	2.748	0.448	9.326	0.872	0.706	<0.001
	4	6.155	56.83	2.905	2.086	0.456	6.285	0.887	0.804	<0.001
ACC2/MIN	1	19.343	198.73	2.808	−1.797	0.441	5.070	0.858	0.866	0.002
	2	20.483	202.56	2.509	0.525	0.414	0.455	0.809	0.975	0.642
	3	20.933	213.12	5.266	2.318	0.448	9.369	0.872	0.781	<0.001
	4	21.591	203.51	4.903	3.454	0.456	14.896	0.887	0.637	<0.001
DEC2/MIN	1	19.278	196.64	2.742	−1.919	0.441	5.634	0.858	0.850	<0.001
	2	20.590	203.49	2.467	0.378	0.414	0.609	0.809	0.980	0.804
	3	21.150	212.50	5.301	2.434	0.448	10.051	0.872	0.771	<0.001
	4	21.626	205.57	4.874	3.340	0.456	14.224	0.887	0.661	<0.001
ACC-DEC/MIN	1	38.621	394.83	5.539	−1.870	0.441	5.398	0.858	0.856	0.001
	2	41.074	404.83	4.951	0.470	0.414	0.564	0.809	0.979	0.770
	3	42.082	425.62	10.561	2.380	0.448	9.734	0.872	0.775	<0.001
	4	43.217	409.08	9.773	3.402	0.456	14.587	0.887	0.650	<0.001
J3G/MIN	1	0.200	0.20	0.165	0.555	0.441	−0.382	0.858	0.933	0.074
	2	0.131	0.09	0.109	0.983	0.414	0.276	0.809	0.890	0.004
	3	0.147	0.12	0.146	1.825	0.448	5.143	0.872	0.830	<0.001
	4	0.133	0.10	0.123	0.462	0.456	−1.268	0.887	0.877	0.005
L5/MIN	1	0.230	0.18	0.189	0.438	0.441	−1.034	0.858	0.913	0.024
	2	0.196	0.21	0.134	0.232	0.414	−0.750	0.809	0.958	0.237
	3	0.177	0.18	0.131	0.326	0.448	−0.607	0.872	0.952	0.236
	4	0.201	0.13	0.208	1.859	0.456	4.283	0.887	0.821	<0.001
IMP8/MIN	1	1.366	11.51	0.940	0.344	0.441	−1.268	0.858	0.928	0.054
	2	1.091	0.91	0.691	0.760	0.414	−0.166	0.809	0.934	0.049
	3	1.054	0.82	0.720	1.049	0.448	0.634	0.872	0.907	0.019
	4	1.060	0.91	0.782	0.960	0.456	0.292	0.887	0.889	0.009
PL/MIN	1	1.363	13.78	0.281	−2.162	0.441	8.149	0.858	0.822	<0.001
	2	1.077	10.41	0.269	−1.155	0.414	6.850	0.809	0.844	<0.001
	3	1.128	11.27	0.158	−0.244	0.448	1.477	0.872	0.961	0.379
	4	1.066	10.01	0.247	1.318	0.456	3.637	0.887	0.898	0.014
HGA/MIN	1	2.359	21.67	1.408	0.179	0.441	−1.308	0.858	0.937	0.091
	2	1.936	16.89	1.134	0.526	0.414	−0.354	0.809	0.967	0.412
	3	1.922	18.46	1.143	1.079	0.448	1.536	0.872	0.929	0.066
	4	1.793	17.95	1.193	0.638	0.456	−0.358	0.887	0.925	0.060
HRAVG	1	153.800	158.00	18.014	−1.341	0.464	2.316	0.902	0.872	0.005
	2	149.897	152.00	20.944	−1.401	0.434	3.039	0.845	0.886	0.005
	3	152.375	154.50	15.542	−0.762	0.472	1.114	0.918	0.938	0.144
	4	149.478	151.00	16.306	−0.761	0.481	0.770	0.935	0.926	0.092
HRMAX	1	178.120	180.000	16.097	−2.190	0.464	7.644	0.902	0.810	<0.001
	2	179.379	184.000	20.365	−2.566	0.434	6.821	0.845	0.679	<0.001
	3	181.875	183.500	10.723	−0.933	0.472	0.789	0.918	0.934	0.117
	4	177.826	179.00	13.885	−1.412	0.481	2.041	0.935	0.853	0.003

Dist/Min = Distance per minute, HIA/Min = High Intensity Actions per Minute, Acc2/Min = Accelerations higher than 2G per minute, Dec2/Min = Decelerations higher than 2G per minute, Acc-Dec/Min = Ratio Acceleration–Deceleration per minute, J3G/Min = Jumps over 3G per minute, L5G/Min = Landing over 5G per minute, HGA/Min = High G Actions per minute, Imp8/Min = Impacts over 8G per minute, PL/Min = Player Load per minute, HRAvg, Average Heart Rate; HRMax, Maximum Heart Rate. Q = quarter.

Across game quarters, the descriptive results indicate relatively stable patterns for both kinematic and neuromuscular variables, with Q1 generally presenting the highest values of the match. Total distance per minute, high-intensity actions, accelerations and decelerations above 2 m s^-2^, jumps and landings over 3G and 5G, impacts per minute, and Player Load per minute all showed their greatest magnitudes in Q1, followed by a moderate reduction in Q2 and Q3 and, in some cases, a partial recovery in Q4. Internal load indicators exhibited a similar pattern, with both average and maximum heart rate reaching slightly higher values in Q1. Overall, these findings suggest that players begin the game with comparatively elevated mechanical and physiological outputs, after which workload stabilizes at slightly lower levels for the remainder of the match.

Table 4 shows that there were significant differences between quarters for the variables DIST/MIN (*F* = 6.508; *p* < 0.001; *partial η* ^*2*^ = 0.172; large effect), PL/MIN (*F* = 6.842; *p* < 0.001; *partial η* ^*2*^ = 0.178; large effect), and HGA/MIN (*F* = 0.923; *p* = 0.038; *partial η* ^*2*^ = 0.030; small effect). No significant differences were observed across quarters for the variables HIA/MIN, ACC2/MIN, DEC2/MIN, ACC-DEC/MIN, J3G/MIN, L5/MIN, IMP8/MIN, HRAVG/MIN, and HRMAX/MIN (*p* > 0.05). However, HIA/MIN did show a significant difference by playing position (*F* = 4.36; *p* = 0.045; *partial η* ^*2*^ = 0.473; large effect).

**TABLE 4:** Fixed effect omnibus tests.

Variable	Comparison	*F*	*Num df*	*Den df*	*p*	*KS - p*	*SW - p*
DIST/MIN	Quarter	6.508	3	94.0	<0.001	0.304	<0.001
	Position	0.599	2	10.9	0.567		
	Quarter ✻ position	0.475	6	93.0	0.826		
HIA/MIN	Quarter	1.60	3	92.82	0.194	0.063	<0.001
	Position	4.36	2	9.70	0.045		
	Quarter ✻ position	1.50	6	91.76	0.188		
ACC2/MIN	Quarter	1.167	3	93.92	0.326	0.040	<0.001
	Position	2.081	2	8.80	0.182		
	Quarter ✻ position	2.219	6	93.02	0.970		
DEC2/MIN	Quarter	1.447	3	94.12	0.234	0.023	<0.001
	Position	2.012	2	9.18	0.189		
	Quarter ✻ position	0.171	6	93.22	0.984		
ACC-DEC/MIN	Quarter	1.304	3	94.03	0.278	0.047	<0.001
	Position	2.049	2	9	0.185		
	Quarter ✻ position	0.194	6	93.13	0.978		
J3G/MIN	Quarter	2.632	3	90.48	0.055	0.225	0.069
	Position	0.784	2	9.14	0.485		
	Quarter ✻ position	1.292	6	89.72	0.269		
L5/MIN	Quarter	0.602	3	94.5	0.615	0.589	0.006
	Position	0.498	2	11.6	0.620		
	Quarter ✻ position	0.297	6	93.6	0.937		
HGA/MIN	Quarter	0.923	3	90.9	0.038	0.16	0.01
	Position	0.894	2	10.2	0.439		
	Quarter ✻ position	1.608	6	90.4	0.154		
IMP8/MIN	Quarter	1.928	3	91.9	0.148	0.145	<0.001
	Position	1.608	2	10.7	0.232		
	Quarter ✻ position	0.794	6	91.2	0.577		
PL/MIN	Quarter	6.842	3	94.6	<0.001	0.199	<0.001
	Position	0.689	2	10.4	0.523		
	Quarter ✻ position	0.449	6	93.7	0.844		
HRAVG	Quarter	0.522	3	82.40	0.668	0.405	<0.001
	Position	1.075	2	9.98	0.378		
	Quarter ✻ position	0.546	6	81.69	0.771		
HRMAX	Quarter	0.231	3	82.8	0.875	0.001	<0.001
	Position	0.608	2	11.1	0.562		
	Quarter ✻ position	0.428	6	82.2	0.858		

Dist/Min = Distance per minute, HIA/Min = High Intensity Actions per Minute, Acc2/Min = Accelerations higher than 2G per minute, Dec2/Min = Decelerations higher than 2G per minute, Acc-Dec/Min = Ratio Acceleration–Deceleration per minute, J3G/Min = Jumps over 3G per minute, L5G/Min = Landing over 5G per minute, HGA/Min = High G Actions per minute, Imp8/Min = Impacts over 8G per minute, PL/Min = Player Load per minute, HRAvg, Average Heart Rate; HRMax, Maximum Heart Rate.

**FIGURE 1:**
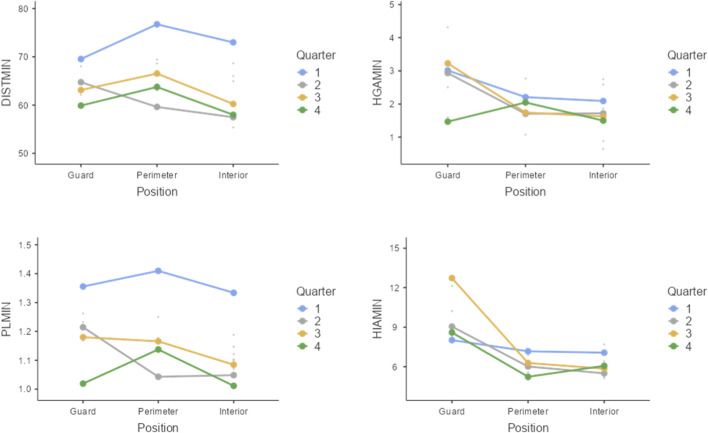
Effects plots. Note: Random effects are plotted by players.

Post hoc analyses using Bonferroni correction (Figure 1) revealed significant differences in DIST/MIN between Q1 and Q2 (*t* = 3.98; *p* < 0.001), Q1 and Q3 (*t* = 2.91; *p* = 0.027), and Q1 and Q4 (*t* = 3.57; *p* = 0.003). For the HGA/MIN variable, values were significantly higher in Q1 compared to Q4 (*t* = 2.91; *p* = 0.027). For PL/MIN, significant differences were found between Q1 and Q2 (*t* = 3.80; *p* = 0.002), Q1 and Q3 (*t* = 2.99; *p* = 0.021), and Q1 and Q4 (*t* = 3.99; *p* < 0.001), with no significant differences observed by position. For the HIA/MIN variable, significant differences were found between guards and centers/power forwards (*t* = 2.75; *p* = 0.047).

Table 5 presents the results of the random effect control for each load variable considering the game quarter and the player’s specific position. When the *conditional R* ^*2*^ is significantly greater than the *marginal R* ^*2*^, this indicates that random effects contribute valuable information, thus justifying the use of a mixed-effects model. In the case of variables such as IMP+8/MIN (0.130 vs. 0.572), HGA/MIN (0.115 vs. 0.660), HRAVG/MIN (0.317 vs. 0.086), and HRMAX/MIN (0.336 vs. 0.061), a strong contribution from random effects is observed.

**TABLE 5:** Linear mixed-effects model based on game quarter and playing position.

Variable	*R* ^*2*^_*marginal*_	*R* ^*2*^_*conditional*_	*AIC*	*BIC*	*ICC*	*LRT*	
DIST/MIN	0.217	0.369	887.815	869.778	0.194	6.93 **	
HIA/MIN	0.205	0.393	572.692	587.862	0.237	8.02 **	
ACC2/MIN	0.088	0.118	650.353	658.973	0.033	0.265	
DEC2/MIN	0.091	0.127	649.832	658.423	0.039	0.382	
ACC-DEC/MIN	0.099	0.123	806.468	798.464	0.036	0.324	
J+3G/MIN	0.018	0.543	−140.957	51.412	0.488	26.8 ***	
L+5/MIN	0.043	0.228	−70.988	12.788	0.193	8.72 **	
HGA/MIN	0.115	0.660	321.953	361.596	0.615	47.8 ***	
IMP+8/MIN	0.130	0.572	231.598	281.445	0.508	39.0 ***	
PL/MIN	0.220	0.278	20.688	95.694	0.074	1.35	0.244
HR_AVG_/MIN	0.086	0.317	874.965	844.656	0.253	10.3 **	
HR_MAX_/MIN	0.061	0.336	848.497	821.086	0.293	15.4 ***	

Dist/Min = Distance per minute, HIA/Min = High Intensity Actions per Minute, Acc2/Min = Accelerations higher than 2G per minute, Dec2/Min = Decelerations higher than 2G per minute, Acc-Dec/Min = Ratio Acceleration–Deceleration per minute, J3G/Min = Jumps over 3G per minute, L5G/Min = Landing over 5G per minute, HGA/Min = High G Actions per minute, Imp8/Min = Impacts over 8G per minute, PL/Min = Player Load per minute, HRAvg, Average Heart Rate; HRMax, Maximum Heart Rate. **p < 0.01, ***p < 0.001.

The *ICC* measures the proportion of total variance attributable to random effects; an *ICC* ≥ 0.1 suggests that random effects (e.g., the player) should be modeled. The *ICC* values for IMP+8/MIN (0.508) and HRMAX/MIN (0.293) indicated a strong influence of the individual player. This effect was even more pronounced for HGA/MIN (0.615), revealing that over 60% of the variance is due to individual differences, highlighting the necessity of using a mixed model. In contrast, ACC2/MIN and DEC2/MIN showed poor model fit, with non-significant low *R* ^*2*^ values indicating that linear mixed models was of limited usefulness for these variables.

## 4 Discussion

The present study aimed to analyze iTL and eTL according to the quarter of the game and the specific playing position of basketball players in real competitive contexts at the professional level. The results confirmed significant variations in certain eTL variables throughout the game, with the first quarter imposing the greatest physical demands. Additionally, relevant differences were identified based on specific playing positions in the variable HIA/MIN, highlighting the necessity to tailor load according to the player’s role. The use of linear mixed models further revealed that a substantial proportion of the variance is attributable to individual factors, thus justifying the appropriateness of this statistical approach. Together, these findings reinforce the importance of individualized and contextualized load monitoring to optimize performance and reduce injury risk (Pernigoni et al., 2021).

Analyzing competition load in real contexts provides essential insights for understanding the physical and physiological demands of professional basketball (Russell et al., 2021). Our findings show that players experienced significant iTL and eTL during official matches, consistent with previous research emphasizing basketball’s high demands in terms of high-intensity actions, repeated impacts, and cardiovascular requirements (Feu et al., 2023), as well as PL/min (Alonso et al., 2023; Pernigoni et al., 2021). Basketball is an intermittent sport in which players alternate prolonged periods of low intensity with intervals of very high intensity (Ibáñez et al., 2023). The fact that average values during competition are so high implies that the worst-case scenarios represent extreme physical demands faced by players (Vázquez-Guerrero and Garcia, 2021). Players must be prepared to transition from very low-intensity situations, such as a stoppage for free throws where they are practically stationary, to very intense moments involving high speed and large accelerations. Strength and conditioning coaches must prepare players during training to optimize their capacity to perform intermittently. These findings highlight the importance of exposing players to training tasks that alternate between prolonged low-intensity phases and abrupt transitions to maximal actions, replicating the oscillatory demands observed in competition. Incorporating drills that combine controlled pacing with sudden accelerations, impacts, and high-speed efforts may help players better tolerate the rapid shifts in intensity characteristic of elite match play.

Regarding load variation by game quarter, significant differences were observed in variables such as distance per minute (DIST/MIN), neuromuscular load (PL/MIN), and high-intensity accelerations (HGA/MIN), with a decreasing trend as the game progressed (Pérez-Chao et al., 2022; Salazar et al., 2024). Understanding the evolution of kinematic and neuromuscular load is essential for implementing strategies to enhance performance and prevent injuries (Ibáñez et al., 2023). Evidently, the first quarter tends to be the most demanding in terms of iTL, with important implications for physical preparation and effort management during competition (Garcia et al., 2022). Several studies have reported higher intensity during the first quarter compared to the last, both in players (García et al., 2020; Garcia et al., 2022; Vazquez-Guerrero et al., 2019; Salazar et al., 2024) and referees (Garcia-Santos et al., 2019). This may be due to factors such as the use of all available timeouts in the last quarter, a higher number of fouls, shorter possession times, and greater fatigue. The magnitude of loads recorded during competition and the differences found depending on the quarter underscore the importance of evaluating not only the total workload of the match but also the quality and intensity at different moments, and even by specific playing position, especially in elite competitions such as the ACB League. From an applied perspective, these patterns suggest that early-game periods may require greater physical readiness and neuromuscular freshness, reinforcing the need to design warm-up protocols and initial rotation strategies that anticipate the heightened demands of the first quarter. Additionally, conditioning programs may benefit from incorporating quarter-specific drills that simulate the progressive reduction in intensity across the game, helping players optimize pacing behaviours and sustain performance under accumulated fatigue.

One possible explanation for the lower eTL observed in professional players, as reflected by reduced HIA/MIN, DIST/min, HGA/MIN, and PL/min, as quarters progress may be an increase in fatigue (Salazar et al., 2024). These authors emphasized that this decrease may be independent of the level of competition, suggesting the need for further studies to objectively determine the causes of this performance decline and/or strategies to minimize it. Conversely, managing the load during the match and for each player, as well as considering the influence of game pace across different periods, constitutes a key decision for coaches who must ensure that the most suitable players are on the court during critical moments of the final quarter, enabling them to achieve higher performance peaks (Pérez-Chao et al., 2021).

Regarding analysis by playing position, significant differences were found in the variable HIA/MIN, demonstrating that high-intensity physical demands vary according to the player’s tactical role (Ibanez et al., 2024; Ferioli et al., 2020). Other studies with professional players have identified differences in multiple eTL and iTL variables depending on position (Gamonales et al., 2023; Puente et al., 2017; Vázquez-Guerrero et al., 2018). For example, Vázquez-Guerrero et al. (2018) found differences in accelerations and decelerations by position, with lower values for perimeter players compared to interior players. Point guards are generally involved in a greater number of explosive actions and short displacements, while centers experience more impacts and perform more eccentric and contact efforts to maintain position and during screens (Stojanovic et al., 2018). High-intensity physical demands vary according to the player’s specific position, reflecting differences in actions such as accelerations, movements, and impacts, which highlights the need for individualized training based on tactical roles. Therefore, strength and conditioning coaches should design specific loads and tasks tailored to the particular demands of each playing position. These positional distinctions reinforce the importance of prescribing conditioning loads that mirror the dominant movement patterns and mechanical stresses associated with each role. Integrating position-specific drills, such as repeated accelerations for guards or high-impact and contact-tolerance tasks for centers, may enhance the specificity and effectiveness of physical preparation throughout the competitive season.

No differences in iTL (HRAVG/MIN and HRMAX/MIN) were found in the present study; however, previous research, such as Puente et al. (2017), reported lower physiological demands in centers compared to guards and forwards. In general, perimeter players tend to cover greater distances and execute actions at higher speeds (Ponce Bordón et al., 2021; Martinho et al., 2025). Overlooking these positional differences may result in suboptimal training design and inappropriate load distribution, especially when a uniform training approach is applied to a heterogeneous group of athletes (Martinho et al., 2025). Although this study did not observe significant differences in iTL, existing evidence suggests that guards and forwards typically experience higher physiological demands than centers. Therefore, implementing a homogeneous training plan may be ineffective, and it is recommended that training loads be adjusted according to the specific demands and characteristics of each playing position. From an applied standpoint, the absence of positional differences in iTL in the present sample does not preclude the need for individualized cardiovascular conditioning, particularly given the consistent evidence showing higher physiological demands for perimeter players. Incorporating role-specific aerobic and anaerobic conditioning profiles may therefore help ensure that each position receives an appropriate stimulus aligned with its typical match demands.

The use of linear mixed models allowed joint evaluation of the effects of game quarter, specific position, and individual (random) player effects. The difference between *marginal and conditional R* ^*2*^ in variables such as IMP+8/MIN, HGA/MIN, and HRAVG/MIN fully justifies this modeling approach, evidencing that a substantial portion of the variance is due to individual factors not explained by fixed effects. Moreover, high *ICC* values (>0.25) in these variables indicated strong player dependency, reinforcing the need to individualize load even within the same position or game situation. This statistical approach aligns with current methodological recommendations for load analysis in invasion sports (Iannaccone et al., 2021). The *ICC* values reflected the influence of innate individual differences on the monitored load variables. Metrics such as high-intensity jumps, actions involving elevated G-forces, and impacts exceeding 8G showed differences, of which at least 50% can be attributed to inter-individual variability. Similar patterns of neuromuscular load differences between athletes have also been observed in other sports, such as handball (Antúnez et al., 2024). It is essential to account for individual differences when planning training sessions, identifying which physical components can be addressed collectively during on-court team practice, and which should be targeted through individualized sessions with the strength and conditioning coach. These results highlight the relevance of monitoring load at the individual level rather than relying solely on group averages, as players with similar roles may respond very differently to identical stimuli. In practice, integrating individualized thresholds, personalized recovery strategies, and athlete-specific conditioning prescriptions may improve the effectiveness of load management throughout the competitive microcycle.

In summary, this study contributes to the understanding of the distribution and variability of competition load in professional basketball, highlighting the influence of the game quarter. Only one load variable showed differences by specific position, indicating the need for further research to consider contextual aspects of competition (preseason, tournaments, playoffs) and the game (offense, defense, winning vs. losing, match balance, playing time) (Garcia et al., 2022; Miró et al., 2024; Sansone et al., 2025; Scanlan et al., 2025). The results reinforce the necessity of individualized contextualized load monitoring, especially in official competitions, considering both the moment of the game and player-specific characteristics to optimize performance and reduce injury risk.

### 4.1 Limitations

This study has several limitations. First, the sample size was small, as it was restricted to three games, and the analysis did not account for more specific tactical roles. Additionally, the study did not examine the mechanisms that may explain variations in load (e.g., fatigue accumulation or tactical adjustments). It should also be noted that no contextual variables, such as score differential, game outcome, opponent quality, or playing venue; were incorporated into the analysis, despite their potential influence on external and internal load patterns. Future research should address these limitations by increasing the sample size, including a greater number of teams, considering competitive and contextual variables, exploring the relationship between load and technical–tactical performance, and incorporating subjective perceptions of effort alongside physiological measures.

### 4.2 Practical applications

The results indicate that the highest physical demands occur at the beginning of the game; however, from an applied standpoint, it is essential that players are prepared not only to perform effectively in the first quarter but also to repeatedly execute high-intensity actions throughout the entire game, particularly in the final minutes, when accumulated fatigue often constrains performance. In this regard, coaches are encouraged to include training tasks that develop the ability to sustain repeated efforts under fatigue, using small-sided games, interval-based drills with incomplete recovery, and targeted blocks placed at the end of training sessions to simulate decision-making and technical execution under fatigue.

Furthermore, the positional differences and high inter-individual variability observed highlight the need for training programs tailored to the specific profile of each player. Guards may benefit from tasks that involve frequent accelerations, changes of direction, and repeated transition actions, whereas forwards and centers require an emphasis on eccentric strength, physical contact, and repeated interior actions performed under fatigue. Integrating these positional needs with the temporal evolution of game demands supports more precise preparation and enhances players’ ability to maintain performance across all phases of competition.

In terms of load management across congested competition schedules, the present findings suggest that coaches should adjust training volume and intensity according to the temporal distribution of match demands. During weeks with two or more games, sessions should prioritize technical–tactical content while reducing the volume of high-impact and high-acceleration actions, as these represent the most fatiguing neuromuscular components identified in competition. Monitoring variables such as impacts/min and PlayerLoad/min on a daily basis can help practitioners detect residual fatigue and adapt microcycle structure, ensuring that players arrive fresh for high-demand periods such as the first quarter and the closing minutes of games.

These results may also assist in designing evidence-based substitution strategies. Since the first quarter concentrates the highest physical outputs and the final quarter reflects performance under accumulated fatigue, coaches may benefit from planning rotations that preserve key players’ ability to perform high-intensity actions late in the match. Substitution timing can be optimized by monitoring individual in-game load trends, particularly in players with higher accelerometry-based loads or greater susceptibility to fatigue. Implementing individualized thresholds for in-game monitoring, whether through live Player Load, impact counts, or high-acceleration events, can support real-time decision-making and help prevent performance decrement or excessive neuromuscular stress.

## 5 Conclusion

This study shows that the physical demands of professional basketball fluctuate markedly across the quarters of play, with the opening quarter consistently imposing the greatest external and neuromuscular loads. Positional differences were most evident in the frequency of high-intensity actions, particularly among point guards compared with centers. The application of linear mixed-effects models highlighted substantial inter-individual variability, underscoring that load behavior cannot be fully explained by group factors alone. These findings emphasize the importance of individualized and context-specific monitoring to capture the true dynamics of competition. Future research should extend this work by examining larger samples, incorporating contextual variables such as match status and tactical phases, and exploring how physical load interacts with technical and tactical performance indicators.

## Data Availability

The raw data supporting the conclusions of this article will be made available by the authors, without undue reservation.

## References

[B1] Alonso E. Trapero J. Ribas C. Sosa C. Gómez M. A. Lorenzo A. (2023). External peak demands are not affected by playing venue (home vs away) during official male basketball games. Int. J. Perform. Analysis Sport 23, 334–342. 10.1080/24748668.2023.2238164

[B2] Antúnez A. López-Sierra P. Vila-Suárez H. Ibáñez S. J. (2024). Neuromuscular load in professional women's handball: segmentation of the player load and the Impacts at group and individual level. Sensors 24 (17), 5750. 10.3390/s24175750 39275660 PMC11398247

[B3] Ato M. López J. J. Benavente A. (2013). A classification system for research designs in psychology. An. Psicol. 29, 1038–1059. 10.6018/analesps.29.3.178511

[B4] Bastida-Castillo A. Gómez-Carmona C. D. De la Cruz-Sánchez E. Reche-Royo X. Ibáñez S. J. Pino-Ortega J. (2019). Accuracy and inter-unit reliability of ultra-wide-band tracking system in indoor exercise. Appl. Sci. 9 (5), 939. 10.3390/app9050939

[B5] Conte D. Palumbo F. Guidotti F. Matulaitis K. Capranica L. Tessitore A. (2022). Investigating external and internal loads in Male older adult basketball players during official games. J. Funct. Morphol. Kinesiol. 7, 111. 10.3390/jfmk7040111 36547657 PMC9782224

[B6] Ferioli D. Rampinini E. Martin M. Rucco D. Torre A. Petway A. (2020). Influence of ball possession and playing position on the physical demands encountered during professional basketball games. Biol. Sport 37, 269–276. 10.5114/biolsport.2020.95638 32879549 PMC7433335

[B7] Feu S. García-Ceberino J. M. López-Sierra P. Ibáñez S. J. (2023). Training to compete: are basketball training loads similar to competition achieved? Appl. Sci. 13 (22), 12512. 10.3390/app132212512

[B8] Field A. (2013). Discovering statistics using SPSS. London: Sage Publications.

[B9] Fox J. L. Scanlan A. T. Stanton R. (2017). A review of player monitoring approaches in basketball: current trends and future directions. J. Strength Cond. Res. 31, 2021–2029. 10.1519/JSC.0000000000001964 28445227

[B10] Fox J. L. Salazar H. Garcia F. Scanlan A. T. (2021). Peak external intensity decreases across quarters during basketball games. Montenegrin J. Sports Sci. Med. 10, 25–29. 10.26773/mjssm.210304

[B11] Gamonales J. M. Hernández-Beltrán V. Escudero-Tena A. Ibáñez S. J. (2023). Analysis of the external and internal load in professional basketball players. Sports 11 (10), 195. 10.3390/sports11100195 37888522 PMC10610899

[B12] García F. Vázquez-Guerrero J. Castellano J. Casals M. Schelling X. (2020). Differences in physical demands between game quarters and playing positions on professional basketball players during official competition. J. Sports Sci. Med. 19, 256–263. 32390718 PMC7196749

[B13] Garcia F. Salazar H. Fox J. L. (2022). Differences in the Most demanding scenarios of basketball match-play between game quarters and playing positions in professional players. Montenegrin J. Sports Sci. Med. 11, 15–28. 10.26773/mjssm.220302

[B14] García F. Schelling X. Castellano J. Martín-García A. Pla F. Vázquez-Guerrero J. (2022). Comparison of the most demanding scenarios during different in-season training sessions and official matches in professional basketball players. Biol. Sport 39, 237–244. 10.5114/biolsport.2022.104064 35309543 PMC8919871

[B15] García-Cuevas P. Pérez-Serrano P. Jiménez-Sáiz S. Bustamante-Sánchez A. (2025). Cuantificación de la carga interna y externa en el baloncesto femenino de élite. Una revisión sistemática. E-balonmano Com, J. Sports Sci. 21, 447–458.

[B16] García-Santos D. Pino-Ortega J. García-Rubio J. Vaquera A. Ibáñez S. J. (2019). Internal and external demands in basketball referees during the U-16 European women's championship. Int. J. Environ. Res. Public Health 16. 10.3390/ijerph16183421 31540097 PMC6765851

[B17] Gómez-Carmona C. D. Bastida-Castillo A. Ibáñez S. J. Pino-Ortega J. (2020). Accelerometry as a method for external workload monitoring in invasion team sports. A systematic review. PLoS One 15, e0236643. 10.1371/journal.pone.0236643 32841239 PMC7447012

[B18] Harriss D. J. Atkinson G. (2015). Ethical standards in sport and exercise science research: 2016 update. Int. J. Sports Med. 36, 1121–1124. 10.1055/s-0035-1565186 26671845

[B19] Iannaccone A. Conte D. Cortis C. Fusco A. (2021). Usefulness of linear mixed-effects models to assess the relationship between objective and subjective internal load in team sports. Int. J. Environ. Res. Public Health 18, 392. 10.3390/ijerph18020392 33419133 PMC7825485

[B20] Ibáñez S. J. López-Sierra P. Lorenzo A. Feu S. (2023). Kinematic and neuromuscular ranges of external loading in professional basketball players during competition. Appl. Sci. 13 (21), 11936. 10.3390/app132111936

[B21] Ibáñez S. J. Gómez-Carmona C. D. López-Sierra P. Feu S. (2024). Intensity thresholds for external workload demands in basketball: is individualization based on playing positions necessary? Sensors 24 (4), 1146. 10.3390/s24041146 38400303 PMC10891821

[B22] Lago C. Lorenzo-Calvo A. Cárdenas D. Alarcón F. Ureña A. Giménez F. J. (2020). La creación de conocimiento en los deportes de equipo. Sobre el tamaño de la muestra y la generalización de los resultados. J. Univers. Mov. Perform. 1, 7–8. 10.17561/jump.n1.e

[B23] López-Sierra P. Reina M. López-Araya S. Ibáñez S. J. (2025). Impact of training on body composition in elite basketball players. E-balonmano Com, J. Sports Sci. 21, 299–312. 10.17398/1885-7019.21.299

[B24] Martinho D. V. Clemente F. M. Ángel-Gómez M. Rebelo A. Field A. Santos C. C. (2025). Physical, physiological, technical and tactical responses according to the playing position in Male basketball: a systematic scoping review. J. Hum. Kinet. 96, 5–35. 10.5114/jhk/203326 40453900 PMC12121883

[B25] Miró A. Vicens-Bordas J. Beato M. Salazar H. Coma J. Pintado C. (2024). Differences in physical demands and player's individual performance between winning and losing quarters on U-18 basketball players during competition. J. Funct. Morphol. Kinesiol. 9 (4), 211. 10.3390/jfmk9040211 39584864 PMC11587006

[B26] Montero I. León O. G. (2007). A guide for naming research studies in psychology. Int. J. Clinical Health Psychology 7, 847–862.

[B27] Pérez-Chao E. A. Lorenzo A. Scanlan A. T. Lisboa P. Sosa C. Gómez M. Á. (2021). Higher playing times accumulated across entire games and prior to intense passages reduce the peak demands reached by elite, junior, male basketball players. Am. J. Men's Health 15 (5), 15579883211054353. 10.1177/15579883211054353 34720014 PMC8558607

[B28] Pérez-Chao E. A. Gómez M. Á. Lisboa P. Trapero J. Jiménez S. L. Lorenzo A. (2022). Fluctuations in external peak demands across quarters during basketball games. Front. Physiol. 13, 868009. 10.3389/fphys.2022.868009 35492582 PMC9039040

[B29] Pernigoni M. Ferioli D. Butautas R. La Torre A. Conte D. (2021). Assessing the external load associated with high-intensity activities recorded during official basketball games. Front. Psychol. 12, 668194. 10.3389/fpsyg.2021.668194 33927675 PMC8076679

[B30] Ponce Bordón J. C. Ramírez Bravo I. López Gajardo M. Á. Díaz García J. (2021). Monitorización de la carga de entrenamiento por posición y tareas en baloncesto profesional masculino. E-balonmano Com, J. Sports Sci. 17, 145–152.

[B31] Puente C. Abian-Vicen J. Areces F. Lopez R. Del Coso J. (2017). Physical and physiological demands of experienced Male basketball players during a competitive game. J. Strength Cond. Res. 31, 956–962. 10.1519/JSC.0000000000001577 27467516

[B32] Reina M. Mancha-Triguero D. Ibáñez S. J. (2022). Monitoring of a competitive microcycle in professional women's basketball through inertial devices. Rev. Int. De. Med. 22, 663–685. 10.15366/rimcafd2022.87.015

[B33] Russell J. L. Mclean B. D. Impellizzeri F. M. Strack D. S. Coutts A. J. (2021). Measuring physical demands in basketball: an explorative systematic review of practices. Sports Med. 51, 81–112. 10.1007/s40279-020-01375-9 33151481

[B34] Salazar H. Ujakovic F. Plesa J. Lorenzo A. Pérez-Chao E. A. (2024). Do elite basketball players maintain peak external demands throughout the entire game? Sensors 24, 4318. 10.3390/s24134318 39001097 PMC11244264

[B35] Sánchez A. Abrunedo J. Caparrós T. (2019). Accelerometry in basketball. Study of external load during training. Apunts Educ. Física Y Deport. 4, 100–117.

[B36] Sánchez-Castillo C. Pons T. C. (2022). External training load by accelerometery analysis during pick and roll in male amateur basketball. E-balonmano Com, J. Sports Sci. 18, 105–116. 10.17398/1885-7019.18.105

[B37] Sansone P. Gasperi L. Tessitore A. Gomez M. A. Gomez M. A. (2021). Training load, recovery and game performance in semiprofessional male basketball: influence of individual characteristics and contextual factors. Biol. Sport 38, 207–217. 10.5114/biolsport.2020.98451 34079165 PMC8139347

[B38] Sansone P. Alonso Perez Chao E. Li F. Gasperi L. Gómez-Ruano M. A. Conte D. (2025). Contextual factors influencing basketball training and competition demands: a systematic review. Int. J. Sports Med. 46 (6), 430–436. 10.1055/a-2533-0917 40090325

[B39] Scanlan A. T. Wen N. Tucker P. S. Dalbo V. J. (2014). The relationships between internal and external training load models during basketball training. J. Strength Cond. Res. 28, 2397–2405. 10.1519/JSC.0000000000000458 24662233

[B40] Scanlan A. T. Power C. J. López-Sierra P. Ibáñez S. J. Elsworthy N. (2025). Everything is not equal – comparisons in sessional training and game loads between main and bench players in a female basketball team. E-balonmano Com, J. Sports Sci. 21, 333–346. 10.17398/1885-7019.21.333

[B41] Staunton C. A. Abt G. Weaving D. Wundersitz D. W. T. (2022). Misuse of the term 'load' in sport and exercise science. J. Sci. Med. Sport 25, 439–444. 10.1016/j.jsams.2021.08.013 34489176

[B42] Stojanović E. Stojiljković N. Scanlan A. T. Dalbo V. J. Berkelmans D. M. Milanović Z. (2018). The activity demands and physiological responses encountered during basketball match-play: a systematic review. Sports Med. 48, 111–135. 10.1007/s40279-017-0794-z 29039018

[B43] Svilar L. Castellano J. Jukic I. Casamichana D. (2018). Positional differences in elite basketball: selecting appropriate training-load measures. Int. J. Sports Physiology Perform. 13, 947–952. 10.1123/ijspp.2017-0534 29345556

[B44] Tuttle M. C. Power C. J. Dalbo V. J. Scanlan A. T. (2024). Intensity zones and intensity thresholds used to quantify external load in competitive basketball: a systematic review. Sports Med. 54, 2571–2596. 10.1007/s40279-024-02058-5 38888854 PMC11467009

[B45] Vázquez-Guerrero J. Garcia F. (2021). Is it enough to use the traditional approach based on average values for basketball physical performance analysis? Eur. J. Sport Sci. 21, 1551–1558. 10.1080/17461391.2020.1838618 33070715

[B46] Vázquez-Guerrero J. Suarez-Arrones L. Casamichana Gómez D. Rodas G. (2018). Comparing external total load, acceleration and deceleration outputs in elite basketball players across positions during match play. Kinesiology 50, 228–234. 10.26582/k.50.2.11

[B47] Vazquez-Guerrero J. Fernandez-Valdes B. Jones B. Moras G. Reche X. Sampaio J. (2019). Changes in physical demands between game quarters of U18 elite official basketball games. PLoS One 14, e0221818. 10.1371/journal.pone.0221818 31479464 PMC6720027

[B48] Wellm D. Jager J. Zentgraf K. (2024). Dismissing the idea that basketball is a “contactless” sport: quantifying contacts during professional gameplay. Front. Sports Act. Living 6, 1419088. 10.3389/fspor.2024.1419088 39108981 PMC11300249

